# Spinal cord ischemia after elective endovascular abdominal aortic aneurysm repair in a patient with multiple occlusions of the intercostal and internal iliac arteries

**DOI:** 10.1016/j.jvscit.2022.06.007

**Published:** 2022-07-09

**Authors:** Yojiro Koda, Katsuhiro Yamanaka, Atsushi Omura, Tomoyuki Gentsu, Masato Yamaguchi, Kenji Okada

**Affiliations:** a Division of Cardiovascular Surgery, Department of Surgery, Kobe University Hospital, Kobe, Hyogo, Japan; b Department of Radiology, Kobe University Hospital, Kobe, Hyogo, Japan

**Keywords:** Endovascular abdominal aortic aneurysm repair, Spinal cord ischemia, Monoplegia, Spinal cord circulation

## Abstract

Spinal cord ischemia (SCI) after endovascular abdominal aortic aneurysm repair is a rare but devastating complication. Occlusion of the artery of Adamkiewicz or feeders to the collateral network for spinal cord circulation (such as the subclavian, intercostal, lumbar, and internal iliac arteries) is associated with the onset of SCI. We present a case of monoplegia owing to SCI after elective endovascular abdominal aortic aneurysm repair with coil embolization of the left internal iliac artery in an elderly patient with a history of arteriosclerosis obliterans and aortic dissection, preoperatively occluding multiple intercostal arteries and the right internal iliac artery.

Endovascular abdominal aortic aneurysm repair (EVAR) is the standard therapy for abdominal aortic aneurysm (AAA) along with open repair^1^^,^^2^ and is favorable for patients with critical risk factors such as old age, malignant tumors, and history of abdominal incision. Spinal cord ischemia (SCI) after EVAR is rare but results in a serious condition that can cause lower limb motor and sensory deficits and vesicorectal disorders. The incidence of SCI after EVAR has been reported to be 0.21%.^3^

In the present study, we report a rare case of monoplegia owing to SCI after elective EVAR, which might have been caused by embolization of the left internal iliac artery (IIA). Written informed consent for publication of their details was obtained from the patient.

## Case report

**Fig 1:**
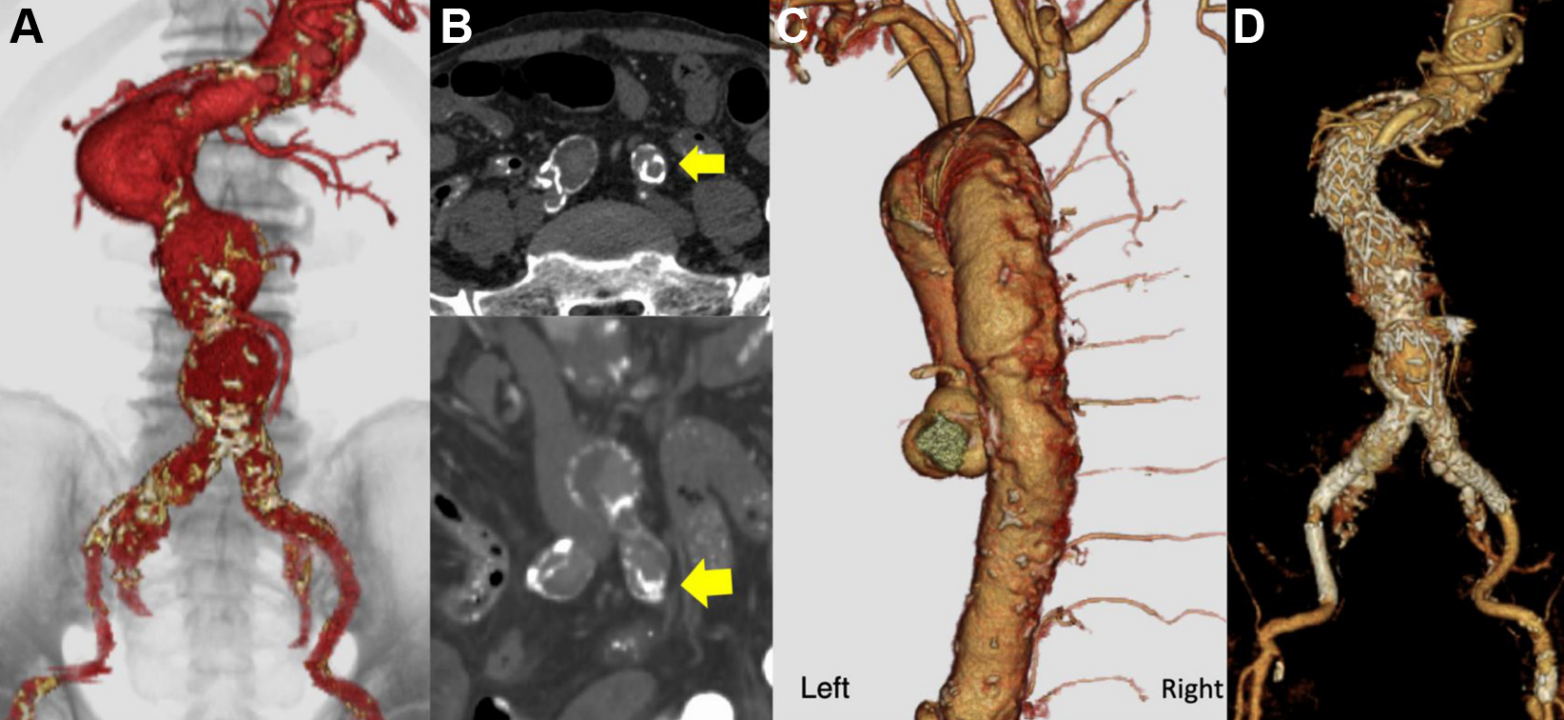
Preoperative and postoperative enhanced computed tomography (CT) findings. **(A)** Abdominal aortic aneurysm (AAA) with three humps. **(B)** Axial (upper) and coronal (lower) CT scan showed the left common iliac artery (CIA) (yellow arrows) with heavily calcification and partial dissection. **(C)** Multiple occlusions of the left intercostal arteries between Th4 and Th12 owing to previous aortic dissection. **(D)** Blood flow in the lower extremities after endovascular AAA repair was confirmed.

The patient was an 89-year-old man who had a history of hypertension, hemodialysis owing to hypertensive nephrosclerosis, chronic obstructive pulmonary disease, bilateral arteriosclerosis obliterans, and type B chronic aortic dissection in the descending aorta and who had undergone percutaneous coronary artery intervention for angina pectoris. He had a clinical frailty score of 3 and was referred to our hospital for EVAR of AAA. Preoperative contrast-enhanced computed tomography (CT) scan revealed an AAA with three humps (55 × 55 mm, 20 × 40 mm, and 20 × 50 mm) (Fig 1, *A*), and patent inferior mesenteric artery (IMA, 5 mm). The length of the left common iliac artery (CIA) was short (12 mm) and heavily calcified with a partial dissection (Fig 1, *B*). In addition, atherosclerotic changes after aortic dissection were found in the posterior wall of the descending thoracic aorta, and the left intercostal arteries were occluded from Th4 to Th12 (Fig 1, *C*).

Elective EVAR was performed under general anesthesia. Bilateral transfemoral access was obtained via surgical cutdown, and 8F sheaths were placed bilaterally in the common iliac arteries. Angiography at the bilateral renal artery level revealed right IIA obstruction. Before EVAR, the left IIA and IMA were selectively catheterized, and embolization was performed using an AMPLATZER VASCULAR PLUG II 12 mm (Abbott, Abbott Park, Chicago, IL) and coils for the left IIA and IMA, respectively. Our indication of IMA embolization is patent IMA. After embolization, the main body of the stent graft (AFX; Endologix, Irvine, CA) was deployed with its proximal end just below the left renal artery. Bilateral external iliac arteries (EIA) were used as distal landing zones, and bare metal stents were added for stenosis in the bilateral EIA. Intraoperative angiography revealed no endoleak. Finally, we performed a bilateral endarterectomy of the bilateral common femoral arteries. The operating time was 325 minutes. Anesthesiologists managed avoid intraoperative and postoperative hypotension, but the patient’s blood pressure fluctuated between 80 mm Hg and 150 mm Hg and was not intentionally maintained at high levels.

**Fig 2:**
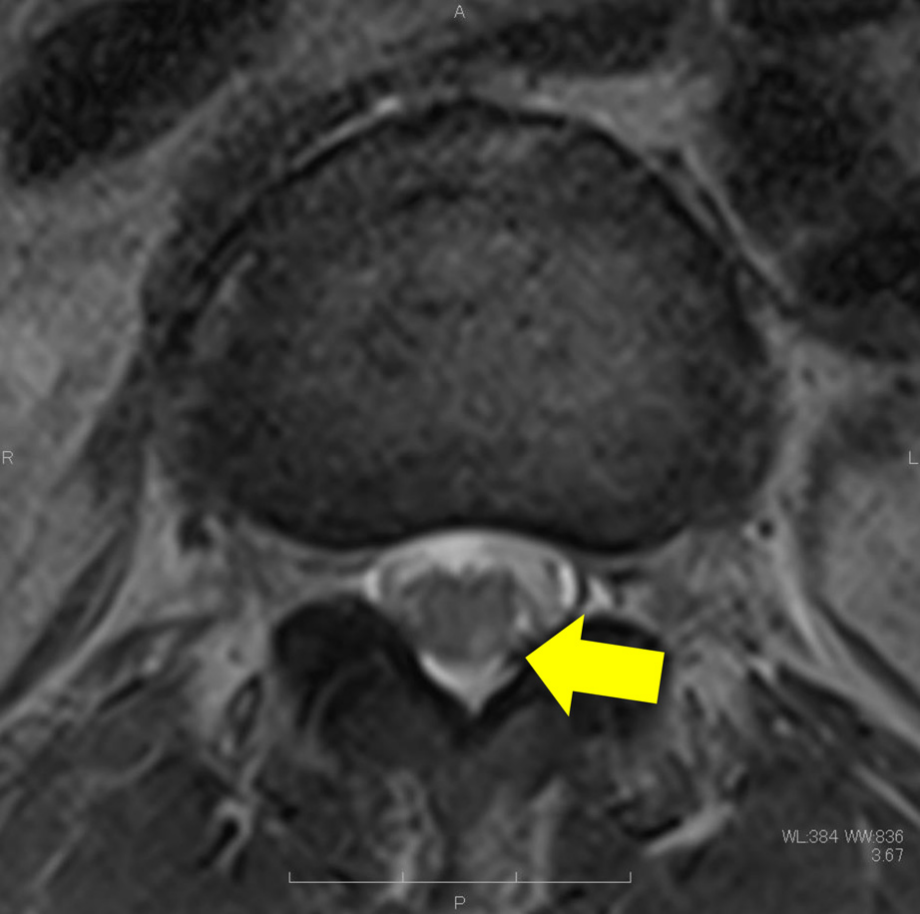
T2-weighted magnetic resonance imaging (MRI) at the L3 level showed a high signal, indicating ischemia, in the left medulla of the lumbar spinal cord (yellow arrow).

On the first day after EVAR, the patient did not complain of sensory or motor disturbance, except for pain in his groin because of the incision. However, on the second postoperative day, sensory disturbance in the left lower leg and motor disturbance in the left thigh and lower leg were apparent. The patient was unable to kneel (manual muscle testing [MMT], 2/5). A contrast-enhanced CT scan showed no stent graft migration or leg kinking, and blood flow in the left lower extremity was normal (Fig 1, *D*). However, there was a high signal in the left medulla at the L3 level on T2 thoracolumbar magnetic resonance imaging (MRI), suggesting ischemia. A neurologist diagnosed monoplegia associated with SCI (Fig 2). After intentional hypertension (systolic blood pressure of >130 mm Hg) and rehabilitation, the strength of the patient’s lower left leg improved (to MMT4). The patient was then transferred to another hospital for rehabilitation. Cerebral spinal fluid drainage (CSFD) was not performed to avoid bleeding complications because he was taking aspirin after the percutaneous coronary artery intervention.

## Discussion

### Mechanism of SCI

The mechanism of SCI is complex and not fully understood. Anatomically, the artery of Adamkiewicz (AKA, defined as the largest anterior radicular artery [between Th8 and L2] feeding anterior spinal artery) is the most critical artery for spinal cord circulation; therefore, occlusion of the AKA (for instance, caused by intraoperative embolization) is considered a main cause of SCI.^4^ However, occlusion of the AKA alone during surgery does not always lead to SCI. The collateral network also plays an important role in blood supply to the spinal cord and may also contribute to instances of SCI. The spinal cord collateral network is formed by feeding arteries such as intercostal arteries other than the AKA, subclavian artery, lumbar arteries, and IIAs,^5^ and occlusion of these can decrease blood supply to the spinal cord. In the present case, we identified occlusion of all left intercostal arteries owing to previous aortic dissection, although the AKA was not identified preoperatively. In addition, preoperative occlusion of the right IIA owing to arteriosclerosis obliterans was observed, and intraoperative embolization of the left IIA was performed. This, rather than occlusion of the AKA, may have led to SCI in this case. Unfortunately, the left CIA in the patient was calcified and narrow with a partial dissection. We could have completed EVAR without IIA embolization if the left CIA was intact.

### Patients’ characteristics suffered from SCI after EVAR

Risk factors for SCI have been reported. Diffuse atherothrombotic aorta (ie, shaggy aorta) is frequently associated with SCI during open repair for thoracoabdominal aortic aneurysm,^6^ and long aortic coverage by thoracic endovascular aortic aneurysm repair increases the risk of SCI.^7^ However, SCI after EVAR has not been well-described. A systematic review mentioned the clinical characteristics of patients with SCI after EVAR, such as complex AAA configuration, multiple procedures, and a long operative time.^8^ In the present case, the patient underwent multiple procedures during EVAR with a long operative time, including left IIA embolization, additional bilateral EIA stenting, and endarterectomy of the bilateral common femoral arteries. The intraoperative systolic blood pressure was between 80 and 150 mm Hg and was not maintained at a high level intentionally.

### Perioperative management for SCI after EVAR

Perioperative preventive management of SCI during EVAR has not been established. However, preemptive maneuvers should be considered in high-risk patients according to this description and the extant SCI management practices during endovascular repair for complex aortic aneurysms.^9^ Although these procedures are not specific to SCI after EVAR, perioperative spinal cord protection practices include blood pressure elevation, hemoglobin level elevation, and CSFD. Rescue maneuvers in patients with suspected SCI include elevating the mean arterial pressure and hemoglobin level, CSFD, and immediate CT scan or MRI. CSFD is an effective procedure against SCI in both prophylactic and rescue situations,^10^ but can cause several complications, such as headache, fever, hematoma in the lumbar region, and subarachnoid hemorrhage (the incidence of these complications is 7.6%-8.1%).^11^^,^^12^ For our patient, an immediate MRI was performed, and high blood pressure was maintained as a rescue maneuver for SCI, but CSFD was not performed because aspirin was administered. Given his multiple vascular occlusions for spinal cord circulation, preoperative spinal cord protection practices, including the identification of acute kidney injury, maintenance of intraoperative and postoperative high blood pressure, postoperative routine MMT testing, and the placement of CSFD, should be considered, although we do not perform perioperative routine management for SCI after elective EVAR. In addition, delayed EVAR, which means EVAR on another day after embolization, may be one of the options to prevent SCI when we need IIA embolization for patient with contralateral IIA occlusion because EVAR results in multiple occlusions of the lumbar artery and may theoretically decrease blood flow to the spinal cord.

## Conclusions

We should pay attention to SCI during elective EVAR in patients with multiple occlusions of the feeding arteries in spinal cord circulation.
